# Association between HLA-DRB1 alleles polymorphism and hepatocellular carcinoma: a meta-analysis

**DOI:** 10.1186/1471-230X-10-145

**Published:** 2010-12-21

**Authors:** Zhong-Hua Lin, Yong-Ning Xin, Quan-Jiang Dong, Qing Wang, Xiang-Jun Jiang, Shu-Hui Zhan, Ying Sun, Shi-Ying Xuan

**Affiliations:** 1 Medical College of Qingdao University, Qingdao 266021, Shandong Province, China; 2 Department of Gastroenterology, Qingdao Municipal Hospital, Qingdao 266021, Shandong Province, China; 3 College of Medicine and Pharmaceutics, Ocean University of China, Qingdao 266003, Shandong Province, China

## Abstract

**Background:**

HLA-DRB1 allele polymorphisms have been reported to be associated with hepatocellular carcinoma susceptibility, but the results of these previous studies have been inconsistent. The purpose of the present study was to explore whether specific HLA-DRB1 alleles (DRB1*07, DRB1*12, DRB1*15) confer susceptibility to hepatocellular carcinoma.

**Methods:**

Case-control studies on HLA-DRB1 alleles association with HCC were searched up to January 2010 through a systematic review of the literature. The odds ratios (ORs) of HLA-DRB1 allele distributions in patients with hepatocellular carcinoma were analyzed against healthy controls. The meta-analysis software REVMAN 5.0 was applied for investigating heterogeneity among individual studies and for summarizing all the studies. Meta-analysis was performed using fixed-effect or random-effect methods, depending on absence or presence of significant heterogeneity.

**Results:**

Eight case-control studies were included in the final analysis. Among the 3 HLA-DRB1 alleles studied, DRB1*07 and DRB1*12 were significantly associated with the risk of HCC in the whole populations (OR = 1.65, 95% CI: 1.08-2.51, P = 0.02 and OR = 1.59, 95% CI: 1.09-2.32, P = 0.02, respectively). No significant association was established for DRB1*15 allele with HCC in the whole populations. Subgroup analysis by ethnicity showed that DRB1*07, DRB1*12 and DRB1*15 alleles significantly increased the risk of hepatocellular carcinoma in Asians (OR = 2.10, 95% CI: 1.06-4.14, P = 0.03; OR = 1.73, 95% CI: 1.17-2.57, P = 0.006 and ***OR ***= 2.88, ***95%CI: 1***.77-4.69, P <***0.001***, respectively).

**Conclusion:**

These results support the hypothesis that specific HLA-DRB1 alleles might influence the susceptibility of hepatocellular carcinoma. Large, multi-ethnic confirmatory and well designed studies are needed to determine the host genetic determinants of hepatocellular carcinoma.

## Background

Hepatocellular carcinoma (HCC) is linked to the interaction between genetic, immunologic, environmental, dietary, and life style factors. Its incidence and distribution vary widely among ethnic groups, sex, and geographic regions. Hepatocellular carcinoma is the third most common cause of cancer-related deaths worldwide with about 600,000 patients dying from the disease annually [1]. Asian countries account for nearly 78% of the roughly 600,000 cases of hepatocellular carcinoma (HCC) reported globally each year [2]. China alone accounts for more than 50% of the world's cases [3]. HBV and HCV infection, liver cirrhosis, male gender, and old age are important risk factors of HCC. The clustering of HCC within families raises the possibility that genetic factors are also involved in susceptibility to HCC.

The Major Histocompatibility Complex (MHC) plays a key role in anti-virus and tumor defense. Human leukocyte antigens (HLA) function in the regulation of immune response to foreign antigens and discrimination of self from non-self antigens. They are encoded by a series of closely linked genetic loci found on chromosome 6 [4,5]. HLA polymorphism is implicated in conferring genetic susceptibility to a large number of immune mediated diseases, including some cancers. Given the pivotal role of HLA molecules in the immune system, several studies have been performed to investigate the association between specific HLA alleles and HCC. However, the association between HLA-DRB1 alleles and HCC in different ethnic populations that has been reported is controversial. Many conflicting reports have been published to date; thus, we performed a systematic review of all of the relevant studies published in the literature to evaluate the association between HLA-DRB1 alleles and HCC. Our principal objectives were to clarify the specific HLA-DRB1 alleles that conferred susceptibility to or which protected against HCC.

## Methods

### Search strategy

Electronic databases (PubMed, EMBASE, Cochrane Library and China National Knowledge Infrastructure) were used to search for all genetic association studies evaluating the HLA-DRB1 polymorphism and HCC in humans in all languages up to January 2010. The search strategy was based on combinations of the terms: HLA-DRB1 AND (Hepatocellular carcinoma or HCC) AND (variants or polymorphism or alleles). We also performed a full manual search from the bibliographies of selected papers. We also contacted the authors of studies containing relevant information, who did not report the results necessary for this analysis. Unpublished data were also accepted if an abstract was available and further information was obtained from the author.

### Selection criteria

In the meta-analysis, the following inclusive selection criteria were set and reviewed by two independent investigators: (1) each trial is an independent case-control study; (2) the purpose of all studies and statistical methods is similar; (3) it supplied enough information to calculate the odds ratio (OR);(4) HLA-DRB1 alleles were molecularly typed (high or low resolution level); (5) inclusion of patients according to the diagnosis standard of HCC defined in 2002, based on at least one of the following criteria: classical histological characteristics or serum a-fetoprotein (AFP) level higher than 400 ng/ml together with radiological findings (ultrasound and/or CT) consistent with HCC [6]. A single study, Donaldson et al, done before 2002, was included in the meta-analysis given that the inclusion criteria of patients were similar to the diagnosis standard. The following exclusive selection criteria were set: (1) incomplete raw data; (2) repetitive reports (if more than one version of the same study was retrieved, only the most recent is used); (3) materials and methods were not well-described and reliable.

Although assessment of study quality is considered important for systematic reviews and meta-analyses, scoring methods have been considered problematic [7] and may not accurately assess the quality measures of interest [8]. Therefore, we used reliability of patient selection, molecular typing method, and statistical analysis method as quality variables.

The frequency of HLA-DRB1 alleles varies according to ethnic and racial background, with some alleles being extremely rare. Therefore, articles were not required to identify all alleles for inclusion.

### Data extraction

The studies were independently evaluated by two researchers. Discrepancies in the evaluations of some studies were resolved by discussion between the reviewers. The following data were collected from each study: authors, publication year, journal, publication type and language, HLA genotyping method, allele genotyped, allele frequencies, numbers of cases and controls, definitions criteria used for HCC, HCC sample description, controls sample description. Allelic frequency was calculated as the number of cases or controls harboring at least one allele type (HLA-DRB1) divided by the total number of chromosomes included in each of the corresponding groups.

### Statistical analysis

The literature review conformed to PRISMA statement standards, and our research fit the minimum set of items for reporting in systematic reviews and meta-analyses (Additional file 1). Heterogeneity was calculated by means of Cochran's Q test (α = 0.05) and Higgins's (*I^2^*) tests. *I^2 ^*values of 25%, 50% and 75% were assigned as low, moderate, and high estimates, respectively. If the results of the Q test had no significant heterogeneity, the Mantel-Haenszel fixed effect model (Peto method) was used for the combination of data; If the results of the Q test had significant heterogeneity, the Dersimonian-Laird random effects model (DL method) were used for the combination of data [9]. A pooled OR was presented as a standard plot with 95% confidence intervals (95%CIs). Meta-analysis was performed using fixed-effect or random-effect methods, depending on ***absence ***or presence of significant ***heterogeneity. ***As a measure of association between HCC and HLA-DRB1 alleles, we combined odds ratios (ORs) with 95% confidence intervals (95%CIs) stratified by gene subtype of patients and controls in a study. Funnel plots and the Egger's regression asymmetry test were used to evaluate publication bias [10]. All *P *values presented are two-tailed. To reduce heterogeneity and to evaluate whether there was a different genotype effect in predefined subgroups of studies, we performed subgroup analysis according to the ethnicity. We performed a sensitivity analysis to assess the stability of the results by sequential omission of individual studies. The analyses were performed using Revman 5.0 provided by the Cochrane Collaboration.

## Results

### Literature assessment

**Figure 1:**
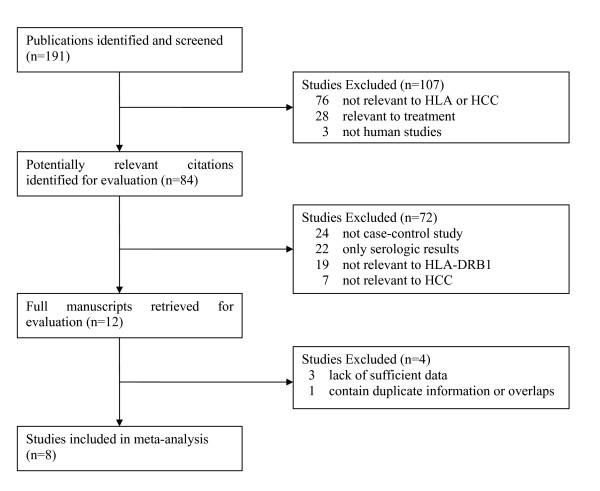
**Flow chart of article selection**.

Figure 1 shows the flow chart of publications identified by the literature search. Search strategy allowed us to identify 84 studies for potential inclusion in the meta-analysis. Finally 8 case-control studies relating to HLA-DRB1 alleles polymorphism and susceptibility to HCC qualified on the basis of our selection criteria [11-18]. A total of 957 subjects were studied (including 341 patients and 616 controls). The main features of the studies included in the meta-analysis are shown in Table 1. Among the eight studies, five studies were conduct in Asian countries; two were conduct in European countries, and one was conduct in African countries. Mean or median age was not stated in 4/8 reports and sex in 4/8. HIV status was determined in only one report [12].

**Table 1:** Characteristics of studies included in the meta-analysis

Author	Year	Country/ Region	Number of HCC (M/F), age	Number of controls (M/F), age	Number of DRB1 alleles studied	HLA genotyping method
Donaldson[11]	2001	Hong Kong	84(79/5),55	124(-/-), NA	13	PCR-SSOP
De Re[12]	2004	Italy	29(-/-), NA	144(-/-), NA	13	PCR-SSP
Yuan[13]^#^	2004	China	10(-/-), NA	50(30/20),43.7 ± 12.9	3	PCR-SSP
López-Vázquez[14]	2004	Spain	46(27/19),62 ± 8	48(19/29),56 ± 12	11	PCR-SSOP
Yuan[15]^#^	2005	China	10(-/-), NA	50(30/20),43.7 ± 12.9	2	PCR-SSP
Kummee[16]	2007	Thai	50(38/12), 57.5 ± 14.2	100(68/32),50.8 ± 13.9	2	PCR-SSP
El-Chennawi[17]	2008	Egypt	50(45/5),51.16 ± 6.16	50(44/6),48.88 ± 9.22	11	PCR-SSP
Pan[18]	2009	China	62(52/10),53.58	50(29/21),30.12	8	PCR-SSP

NA, not available; PCR-SSOP, PCR-sequence-specific oligonucleotides probes; PCR-SSP, PCR-sequence-specific primer;

# These two studies described different allele polymorphisms with the same subjects.

HLA-DRB1 alleles were molecularly typed (high or low resolution level). Five studies used low resolution molecular typing for HLA, while three used high resolution molecular typing for HLA. Low resolution molecular typing methods for HLA could not identify the specific alleles. Accurate methods for HLA class II typing should involve the combination of PCR-SSOP, PCR-SSP, and PCR-SSCP [19].

### Meta-analysis: Association between HLA-DRB1 alleles with HCC

**Figure 2:**
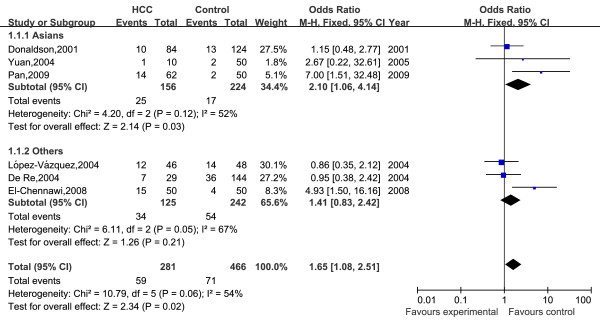
Meta-analysis forest plot of included studies on the association between HLA-DRB1*07 allele and HCC. Each plot shows the effect size and precision for individual studies and for the combined effect. Filled squares are proportional in size to study weights.

**Figure 3:**
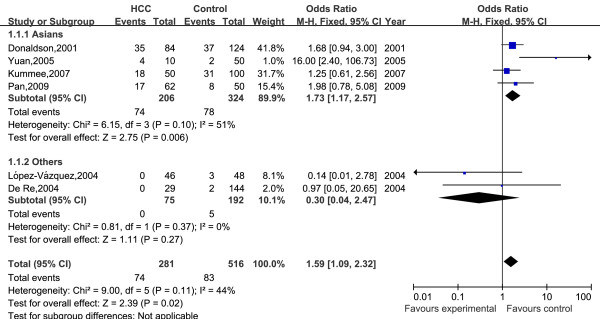
Meta-analysis forest plot of included studies on the association between HLA-DRB1*12 allele and HCC. Each plot shows the effect size and precision for individual studies and for the combined effect. Filled squares are proportional in size to study weights.

**Figure 4:**
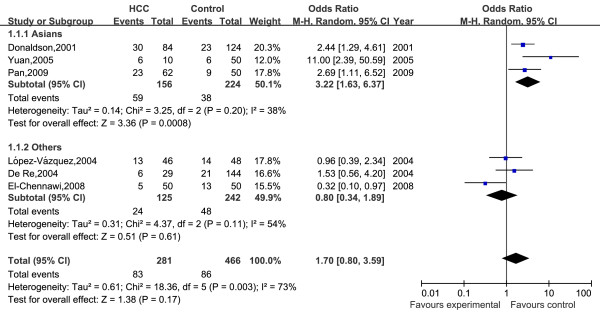
Meta-analysis forest plot of included studies on the association between HLA-DRB1*15 allele and HCC. Each plot shows the effect size and precision for individual studies and for the combined effect. Filled squares are proportional in size to study weights.

A total of 13 HLA-DRB1 alleles were studied in the 8 case-control studies, but only 3 alleles (DRB1*07, DRB1*12, DRB1*15) was extracted from the studies to investigate their association with HCC, which were reported at least six of the eight case-control studies. Statistics calculated for each study are shown in the forest plot (Figures 2, 3 and 4).

In the meta-analysis, overall the frequencies of HLA-DRB1*07 allele was 20.1% (59 of 281) in HCC and 15.2% (71 of 466) in controls. The heterogeneity test indicates that the variation of trial-specific ORs was not statistically significant (χ^2 ^= 10.79, *I^2 ^*= 54%, P = 0.06 and >0.05). Under the fixed effect model, the combined OR for the association of HLA-DRB1*07 allele with the risk for HCC in the whole populations was determined to be 1.65 (95% CI: 1.08-2.51; p = 0.02), and under the random effects model was 1.77 (95%CI: 0.88-3.56; p = 0.11). In sensitivity analysis, the exclusion of individual studies did not change this significant result, except for exclusion of the study by Pan et al and El-Chennawi et al, which produced a non-significant association. Subgroup analysis by ethnicity showed that HLA-DRB1*07 allele significantly increased the risk of hepatocellular carcinoma in Asians under the fixed effect model (OR = 2.10, 95% CI: 1.06-4.14, P = 0.03).

Overall the frequencies of HLA-DRB1*12 allele was 26.3% (74 of 281) in HCC and 16.1% (83 of 516) in controls. The heterogeneity test indicates that the variation of trial-specific ORs was not statistically significant (χ^2 ^= 9.00, *I^2 ^*= 44%, P = 0.11 and >0.05), so the fixed-effect method was used to combine the results. The combined OR for the association of HLA-DRB1*12 allele with the risk for HCC in the whole populations was determined to be 1.59 (95% CI: 1.09-2.32), and was statistically significant (P = 0.02 and <0.05). In sensitivity analysis, the exclusion of individual studies did not change this significant result, except for exclusion of the study by Donaldson, et al and Sun, et al, which produced a non-significant association. Subgroup analysis by ethnicity showed that HLA-DRB1*12 allele significantly increased the risk of hepatocellular carcinoma in Asians (OR = 1.73, 95% CI: 1.17-2.57, P = 0.006).

Meta-analysis for HLA-DRB1*15 allele was carried out, but it did not show any statistical effect in the whole populations. Subgroup analysis by ethnicity showed that HLA-DRB1*15 allele significantly increased the risk of hepatocellular carcinoma in Asians under the fixed effect model (***OR ***= 2.88, ***95% CI: 1***.77-4.69, ***P < 0.001***).

**Figure 5:**
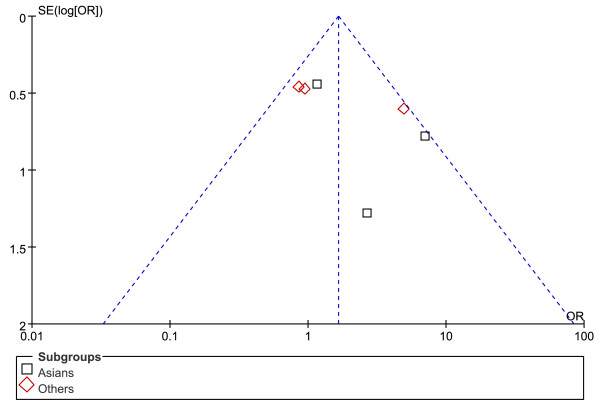
Funnel plot of HLA-DRB1*07 allele and HCC to explore publication bias. OR = odds ratio; SE = standard error.

**Figure 6:**
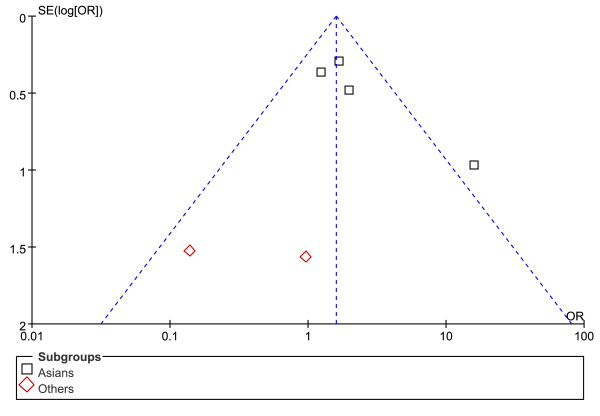
Funnel plot of HLA-DRB1*12 allele and HCC to explore publication bias. OR = odds ratio; SE = standard error.

**Figure 7:**
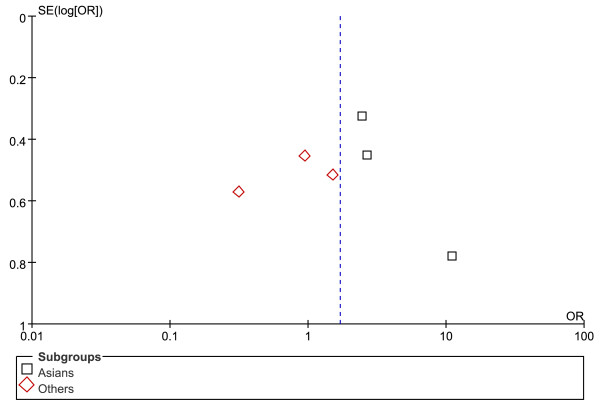
Funnel plot of HLA-DRB1*15 allele and HCC to explore publication bias. OR = odds ratio; SE = standard error.

Figures 5, 6 and 7 show the funnel plot analysis to detect publication bias of each study for DRB1*07, DRB1*12 and DRB1*15, respectively. The shape of the funnel plot seemed to be asymmetrical, suggesting that publication bias might affect the findings of our meta-analysis.

## Discussion

The aim of the present study was to ascertain whether specific HLA-DRB1 alleles are associated with the development/protection of HCC. We analyzed the published studies investigating the association between HLA-DRB1 alleles and HCC. Studies concerning this possible association have been undertaken since the early 1996s [20].

To our knowledge, this is the first published meta-analysis investigating this association. Our meta-analysis of eight studies revealed that DRB1*07 and DRB1*12 were significantly associated with the risk of HCC in the whole populations (OR = 1.65, 95% CI: 1.08-2.51, P = 0.02 and OR = 1.59, 95% CI: 1.09-2.32, P = 0.02, respectively). Subgroup analysis by ethnicity showed that DRB1*07 and DRB1*12 alleles also significantly increased the risk of hepatocellular carcinoma in Asians (OR = 2.10, 95% CI: 1.06-4.14, P = 0.03; OR = 1.73, 95% CI: 1.17-2.57, P = 0.006, respectively). No significant association was established for DRB1*15 allele with HCC in the whole populations. Subgroup analysis by ethnicity showed that DRB1*15 alleles significantly increased the risk of hepatocellular carcinoma in Asians under the fixed effect model (***OR ***= 2.88, ***95% CI: 1***.77-4.69, ***P < 0.001***).

Epidemiological survey showed that Asian countries account for nearly 78% of hepatocellular carcinoma (HCC) reported globally each year, and Hepatitis B Virus (HBV) is the major etiology of HCC in these areas. Although HBV infection plays an important role in HCC, HBV infection alone is not sufficient for progression to HCC. Several lines of evidence suggest that cellular immune surveillance is important in the control of HBV infection and the development of HCC. In 2007, Yang and his colleagues found that HLA-DRB1*07 were markedly higher in the HBV-infected group among people in northwestern China (17.6% of HBV-infected patients vs 9.3% of spontaneously cleared controls, OR = 2.09, P < 0.05) [21]. In 2006, Zhang and his colleagues found that the frequency of HLA-DRB1*12 was significantly higher in the HBV persistent group than in the recovered group among Chinese (0.230 versus 0.063, P = 0.004, OR = 2.09) [22]. In 2003, Amarapurpar and his colleagues found that a positive association of HLA-DRB1*15 to persistence of HBV among Indians (57.6 vs. 25%) [23]. As we know that clearance of acute hepatitis B virus (HBV) infection is associated with a vigorous CD4+ T-cell response focusing on the core protein. HLA class II glycoproteins present viral peptides to CD4+ T cells and influence the immune responses. Binding affinities of overlapping peptides covering the core and envelope proteins of HBV were measured to HLA glycoproteins encoded by some HLA-DRB1 molecules and compared with published peptide-specific CD4+ T-cell responses [24]. So we have a hypothesis that HLA-DRB1*07, DRB1*12 and DRB1*15 alleles may be the key host factors to determine the development of diseases from HBV infection to HCC in Asians, basing on our results that HLA-DRB1*07, DRB1*12 and DRB1*15 alleles significantly increased the risk of hepatocellular carcinoma in Asians. Furthermore, the importance of environmental factors and gene-environmental interactions in the development of HCC should not be ignored and is beginning to be delineated.

Moreover, HLA-DRB1 alleles polymorphism have been reported to be associated with other cancers and autoimmune diseases, including cervical squamous cell carcinoma, rheumatoid arthritis, systemic lupus erythematosus, autoimmune hepatitis, inflammatory bowel disease, multiple sclerosis and type 1 diabetes, and meta-analyses have been done for these diseases [25-31].

Additionally, because the information used in our research was based on data from observational studies, some limitations should be discussed in this meta-analysis and the results should be considered with caution. A primary cause for the difference in results by different authors may be related to the great variability of the frequency of HLA alleles in different populations. It is quite possible that one ethnic group may have some specific alleles in development/protection of HCC compared to other ethnic groups. Many studies were conducted on relatively smaller samples. Insufficient number of individuals might decrease the power to detect a difference in the distribution of DRB1*07, DRB1*12 and DRB1*15 alleles between HCC patients and controls, though a true difference exist. Lack of an association may not mean that associations do not exist. Many studies did not control for the matching variables in the analysis, and the possible confounders could be among the potential causes of variation in the studies' estimates. Different types of control groups could also be among the potential causes of variation in the studies' estimates. Effects of interactions in other environmental/behavioral and/or viral factors may be inevitable. A complex interplay between various genes is likely to modulate the development of HCC rather than a single allele. HLA genotyping techniques must be taken into consideration because these methodologies have different sensitivities. HLA genotyping were mainly tested for by PCR with probe hybridization, but targets were not always verified by sequencing. This could hinder an effective comparison between the studies and influence the combined results. The shape of the funnel plot seemed to be asymmetrical, suggesting that publication bias might affect the findings of our meta-analysis. Furthermore, although we tried to maximize our efforts to identify all relevant published studies in peer-reviewed journals, it is possible that some escaped our attention.

In spite of these, our meta-analysis also had some advantages. First, substantial number of cases and controls were pooled from different studies, which significantly increased statistical power of the analysis. Second, the quality of case-control studies included in the current meta-analysis was satisfactory based on our selection criteria. Third, the patients were phenotypic homogeneous subjects, diagnosed either by gold standard or by biochemical and imageology combined methods, which could reduce the heterogeneity in some extent.

## Conclusion

Our meta-analysis suggests that HLA-DRB1*07 and DRB1*12 alleles are risk factors for HCC in the whole populations, especially in the Asians; DRB1*15 allele is only associated with an increased risk of HCC in Asians (under the fixed effect model). However, it is necessary to conduct large trials using standardized unbiased methods, homogeneous HCC patients and well matched controls, with the assessors blinded to the data. Moreover, gene-gene and gene-environment interactions should also be considered in the analysis. Such studies taking these factors into account may eventually lead to a better, more comprehensive understanding of the association between HLA-DRB1 polymorphism and HCC.

## Abbreviations

HLA: human leukocyte antigens; HCC: hepatocellular carcinoma; HBV: hepatitis B virus; OR: odds ratio; CI: confidence interval.

## Competing interests

The authors declare that they have no competing interests.

## Authors' contributions

ZHL and YNX carried out the design of this meta-analysis, conducted a searching, extracted data, analyzed the data and drafted the manuscript. SYX participated in study design and the critical revision of the manuscript. QJD, QW, SHZ, XJJ and YS participated in the critical revision of the manuscript. All authors read and approved the final manuscript.

## Pre-publication history

The pre-publication history for this paper can be accessed here:

http://www.biomedcentral.com/1471-230X/10/145/prepub

## Supplementary Material

Additional file 1 **PRISMA 2009 Checklist**. We conformed to PRISMA statement standards and provided the detailed information. Click here for file
